# Demographic Strengthening of European Identity

**DOI:** 10.1111/j.1728-4457.2016.00133.x

**Published:** 2016-06-02

**Authors:** Erich Striessnig, Wolfgang Lutz

The european union is currently undergoing a major crisis within its institutions. Many observers think the EU is doomed, particularly in view of the current refugee situation, when in many countries the national agenda overrides the European project and the aggregate result is widely perceived as a failure of European‐level governance. This skepticism is heightened by the UK referendum on continued EU membership. But few of these observers, focused on short‐term political developments and crises, offer any long‐term view of the alternatives. Is it realistic to return to the old system of nation states with closed borders and individual currencies? And does a majority of the European population want such an outcome?

Being critical of current transnational institutional arrangements does not necessarily imply that the citizens of Europe see no future for continued, or possibly even strengthened European‐level collaboration and integration. As Zielonka (2014) states, citizens who have lost trust in the EU are not necessarily happy with the performance of their nation states either. Going back to a pre‐EU era might be even less desirable to them than the present situation. This is also confirmed by a recent Europe‐wide Bertelsmann Study (De Vries and Hoffmann 2015) that finds that people continue to support the idea of a united Europe and do not see nation states as a viable alternative.

The empirical evidence presented in this note suggests that the European population continues to become more European‐minded, despite the continuing economic and political predicaments. Applying the demographic tools of cohort analysis to the responses provided by recent Eurobarometer surveys on expressed national versus European identity, we revisit previous forecasts of the spread of European identity as they were published in 2006 under the title “The demography of growing European identity” (Lutz, Kritzinger, and Skirbekk 2006) and compare them with new empirical evidence for 2013. While we find that some elements of the earlier predictions proved to be off‐base, the overall predictive power of our underlying demographic metabolism model appears to be sound.

## Demographic metabolism at work

The conceptual foundations for the model of demographic metabolism were laid by Norman Ryder, who first introduced the term in his influential paper on “The cohort as a concept in the study of social change” (Ryder 1965). In that article, Ryder defined demographic metabolism as “a massive process of personnel replacement” (p. 843) driven by the births, lives, and deaths of individuals. But while individuals die, societies become immortal if reproduction is sufficient to offset mortality. Ryder combined this thought with the assumption that the individual's flexibility to change is restricted once certain characteristics or attitudes are established. Consequently, he viewed the continuous emergence of new participants in the social process and the withdrawal of their predecessors as the main forces of social transformation. Based on the assumed inflexibility of individuals over their lifetimes, Ryder concluded that “The society whose members were immortal would resemble a stagnant pond” (p. 844).

Lutz (2013) goes beyond Ryder in relaxing the assumption of strong cohort determinism. This step was inspired by developments in the field of demography, particularly multi‐dimensional cohort component analysis (Keyfitz 1985; Rogers 1975), which provided the tools to model changes over the life course. Hence, the possibility of lifelong learning and changes (transitions to other states) within birth cohorts can in itself represent a force of socioeconomic change, such that immortality would no longer necessarily result in Ryder's “stagnant pond.” A second step by Lutz was to argue that, by projecting observable differentials in relevant dimensions of social heterogeneity along cohort lines and over time, multi‐dimensional cohort component analysis can be used to make quantitative forecasts of the future.

To what degree cohort effects dominate age and period effects depends on the specific characteristic studied. In some cases (e.g., highest educational attainment after a certain age) the characteristic is “sticky” by definition—invariant after a certain age (Lutz, Butz, and KC 2014; Lutz and KC 2011). For other, “softer” characteristics (such as self‐assessed European identity), the relative strength of cohort effects is a matter of empirical analysis for the past and corresponding assumptions for the future. If there is indeed a strong cohort effect, then it lends itself to projections along cohort lines, making it a good candidate for the application of the demographic metabolism model.

## Assessing the prevalence of European identity

There are numerous studies in political science on expressed national and multi‐national identities. The identification of citizens with a political system is a necessary precondition for the system's stability and legitimacy (Deutsch 1953). Yet the development of a European identity need not be associated with a decline in specific national and regional identities. Indeed, rather than displacing them, European identity has been shown to complement national and regional identities (Risse 2000). In this way, “national identity is the springboard, not the gravedigger, of European identity, with national identity providing a model of what it is to belong to a remote political community” (Duchesne and Frognier 1995, p. 194).

Identity also reflects the emotional attachment of citizens to a political system. This emotional attachment is the outcome of a process of trust, a socialization process in which norms and values are communicated (Easton 1965) and individuals learn to see themselves as members of their respective “imagined communities” (Anderson 1991). Identity differs from utilitarian allegiance, which focuses on short‐term costs and benefits emanating from the political system. It is entirely possible to observe a decrease in one dimension (e.g., support for EU fiscal policy or even membership) contemporaneous with an increase in the other (e.g., European identity).

To account for these two separate aspects, the Eurobarometer Survey (EB) regularly interviews representative members of European national populations, asking both about people's support for and perceived benefits from EU membership, thus measuring the utilitarian dimension, and about their identity, addressing the affective dimension. The relevant question on European identity was asked several times in identical form, namely: “In the near future, do you see yourself as [Nationality] only, as [Nationality] and European, as European and [Nationality] or European only?” Since 1996, this question has been asked with unchanged wording more than a dozen times in the EU‐15 (members of the EU as of 1995) with national samples of around 1,000 in each round.

In 1996–2004, on average, 42 percent of the adult population of the EU‐15 above age 18 identified themselves solely as nationals of their own country, whereas 58 percent gave an answer that reflected multiple identities including a European identity (see Table 1). According to the 2013 survey, the latest year for which data are available, this latter figure has risen to 61 percent. But this weighted average masks important country differences. While little has changed at the top and bottom of the country list, with European identity seemingly having reached its high point in Luxembourg and Italy already at the turn of the new millennium and the United Kingdom falling even further behind, there are also some notable differences. Ireland has experienced the strongest drop in the prevalence of multiple identity and has moved from the middle of the ranking down to second‐to‐last position, though still well ahead of the UK. Much more important for the population‐weighted average, France has experienced the second largest decline, although from a much higher initial level. Apart from those declines, percentages have gone up in all other countries, with the increase being most notable in Germany, Austria, Sweden, and Finland, but quite surprisingly also in Greece and to a lesser extent other Mediterranean countries where the institutional crisis has had especially negative effects.

**Table 1:** Prevalence of multiple identities within EU‐15, average of 1996–2004 and 2013

Country	1996–2004	2013
Luxembourg	78	77
Italy	72	70
France	68	62
Spain	64	66
Belgium	59	68
Netherlands	59	67
Germany	56	70
Denmark	54	57
Ireland	53	46
Austria	51	64
Portugal	50	54
Greece	46	56
Sweden	45	57
Finland	43	54
UK	40	38
Average (unweighted)	56	60
Average (population weighted)	58	61

SOURCE: Eurobarometer.

**Figure 1:**
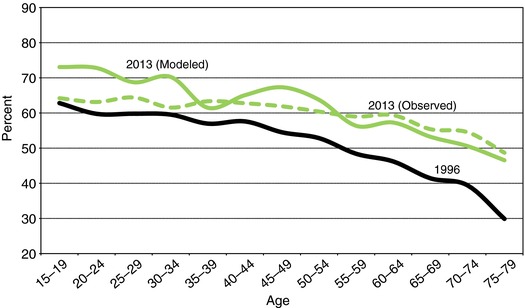
Proportions of EU‐15 population aged 15 and above with multiple identities, by age group, as derived from Eurobarometer Surveys in 1996, projected to 2013 (based on Lutz et al. 2006), and observed in 2013

When plotting the data against age (see curve for 1996 in Figure 1), we observe a clear negative association between having multiple identities and age. The older the respondent, the greater is the chance that he or she will hold only a national identity. While for younger age groups, those with only national identities are a minority, for the population above age 60 they constitute a majority. Can we conclude from this obvious age pattern that as people get older, they develop a stronger national identity and abandon the multiple identities they might have had earlier? If this dominance of an age effect were real, the massive population aging that will occur over the coming decades would suggest a decline in the proportion of Europeans with multiple identities. On the other hand, the same pattern could also be interpreted in terms of a cohort effect: young cohorts being socialized in a way that produces a higher prevalence of multiple identities than among the older cohorts, which they then maintain throughout their lives. This outcome would suggest increases in future European identity through demographic metabolism, with the younger, more European‐minded cohorts replacing the previous, more nationalistic ones.

While both of these contrasting interpretations are plausible, their validity cannot be assessed empirically on the basis of a single cross‐sectional survey. Only panel data that provide age profiles at different points in time allow us to distinguish between age and cohort effects. Using data from 1996 to 2004, Lutz, Kritzinger, and Skirbekk (2006) found evidence of a positive cohort effect indicative of a trend toward greater prevalence of multiple identities in the EU. In other words, cohorts born more recently are socialized in a way that decreases their association with solely national identities and increases their association with multiple identities. These identities were then assumed to be maintained throughout their lives, while for the subsequent younger cohorts, for which no empirical data were available, the authors assumed the continuation of the time trend of past inter‐cohort increases in multiple identities. Based on this model of demographic metabolism, they predicted that by 2030 the proportions with European identity would exceed 70 percent for younger people (below age 45) and 50 percent even for older ones. In terms of absolute numbers, the results predicted that only 104 million adult EU‐15 citizens will have solely national identities in 2030, while 226 million will have multiple identities. Nearly ten years after that prediction was made, a turbulent period for the EU, how closely is it being borne out?

The top two lines in Figure 1 show the age pattern of European identity in 2013 as projected in the 2006 paper (solid) and the actual pattern observed in the 2013 EB survey (dashed). Considering broad age groups, this empirical test of past predictions shows that for cohorts above age 35 in 2013 the forecast was fairly accurate, while for the younger age groups the forecast was too high. For the age groups 15–24 the actual proportions with multiple identities were 8–9 percentage points lower than the forecasted ones. This outcome shows that the assumed continuation to 2013 of the period trend in inter‐cohort increases observed for the 1996–2004 period did not hold. For the new cohorts that entered young adult ages during the projection period, the increasing trend stalled. However, for adults of higher ages the assumption of stability of identities along cohort lines essentially held. There is some noise around the specific smaller age groups, but taken together the European population above age 35 in 2013 shows almost exactly the proportion with European identity (57.9 percent) that was projected on the basis of 1996–2004 data using the demographic metabolism model (58.6 percent). This outcome confirms the predictive power of our approach even during turbulent times.

## Discussion

These findings suggest that the relentless forces of cohort replacement, through which younger and (up to the most recent cohorts) more European‐minded cohorts gradually take the place of older, more nationally oriented cohorts, produce significant and predictable changes in the prevalence of European identity. Because the population above age 35 constitutes a majority of the total European electorate (and exerts disproportionate influence as decisionmakers and politicians), these predictable changes in the composition of the adult population will likely have important long‐term implications for fundamental political and economic developments in Europe, even though short‐term politics are likely to remain volatile. Or, to put it succinctly, despite current problems, do not count Europe out.

These findings are consistent with those of the aforementioned study by the Bertelsmann Foundation (De Vries and Hoffmann 2015) which finds that, contrary to media reports and common opinion, Europe's citizens—with the exception of the British—cannot be classified as Eurosceptics, but rather have merely ambivalent attitudes toward Europe. While the majority of people, even in the economically troubled Mediterranean, support their countries’ membership in the EU and its monetary union (the Eurozone), they are dissatisfied with the EU's current policy direction. The study also finds that even though people are dissatisfied with the current state of affairs in Brussels, they are equally unhappy with the situation in their own national capitals. When it comes to the future functions of the EU, the study finds that both the youngest and the oldest generations favor an EU that safeguards peace and security over one that promotes growth.

At the level of methodology, the important lesson from this exercise in forecast‐checking is that, in contrast to stable cohort patterns, period effects determining the formation of the younger‐cohort identities can go both ways—that is, there is no guarantee of a continuation of previously observed period trends. While the firmly established identities of middle‐ and older‐age adults have not changed much in response to the crises of European institutions, these events have interrupted identity‐formation trends among the younger cohorts. This leaves us with a clear message for forecasting social trends in general: population‐wide trend extrapolation is a much less reliable approach to anticipating future developments than decomposing the population into cohorts, critically assessing the stability of patterns along cohort lines, and then making projections using a demographic metabolism approach.
